# Prevalence and associated factors of scabies among schoolchildren in Dabat district, northwest Ethiopia, 2018

**DOI:** 10.1186/s12199-019-0824-6

**Published:** 2019-11-30

**Authors:** Henok Dagne, Awrajaw Dessie, Bikes Destaw, Walelegn Worku Yallew, Zemichael Gizaw

**Affiliations:** Department of Environmental and Occupational Health and Safety, Institute of Public Health, University of Gondar, Gondar, Ethiopia

**Keywords:** Human scabies, Prevalence, Lesions, Physical examination, Ethiopia

## Abstract

**Background:**

Scabies is a skin infestation caused by the mite *Sarcoptes scabiei* that causes a pruritic skin eruption. In Ethiopia, the disease is common especially during natural or manmade disasters such as flooding, drought, civil war and conflict, poor water supply and sanitation, and overcrowding living condition. Though scabies is becoming a major public health problem, especially in low resource settings, there has been no study that reported the prevalence of scabies in the study area. The aim of this study, therefore, was to assess the prevalence of scabies and associated factors among students in primary schools in Dabat district, northwest Ethiopia.

**Method:**

An institutional-based cross-sectional study was employed to determine the prevalence of scabies and associated factors among schoolchildren in Dabat district. A total of 494 students selected by a multi-stage sampling technique were included in this study. Scabies was diagnosed by medical practitioners based on lesions observed on body surfaces after a physical examination. Bivariable and multivariable binary logistic regression analyses were performed using SPSS version 20. Significance level was obtained at *p* value < 0.05.

**Result:**

The prevalence of scabies was 9.3% (46/494) with 95% CI (5.66%, 12.94%). Among schoolchildren who were infested by scabies, 65.22% (30/46) had a mild, 28.26% (13/46) had moderate, and 6.52% (3/46) had severe lesions. Studying at a rural school (AOR = 2.99, 95% CI 1.33, 6.71), had illiterate father (AOR = 5.11, 95% CI 2.25, 11.58), being grade level 1–4 (AOR = 3.91, 95% CI 1.69, 9.05), rarely taking a bath (AOR = 3.54, 95% CI 1.36, 9.25), contact with a person with itching symptom (AOR = 2.66, 95% CI 1. 21, 5.83), a family member with itchy symptoms (AOR = 4.76, 95% CI 2.20, 10.28), not living with both parents (AOR = 2.49, 95% CI 1.02, 6.06), and using water only for hand washing (AOR = 4.38, 95% CI 1.78, 10.76) were factors associated with scabies infestation among schoolchildren.

**Conclusion:**

The prevalence of human scabies among schoolchildren in Dabat district northwest Ethiopia was high. The school localization, first cycle level of education, paternal educational status, frequency of taking a bath, and contact with a person having itchy lesions, presence of a family member with itchy lesion, and type of frequently used hand washing material were the factors significantly impacting the occurrence of scabies. Special attention should be given to students at first cycle education as they are at the highest risk of infestation.

## Background

Scabies is a skin infestation caused by the mite *Sarcoptes scabiei* that causes a pruritic skin eruption [1]. This skin disease affects more than 300 million people per year worldwide, resulting in considerable morbidity, especially in resource-poor countries [2–4] Scabies is a neglected disease and more common in tropical, humid regions [5].

Evidence from the literature shows that the prevalence of scabies in African countries is persistently high, being as such noticeable among individuals, and in some specific groups and communities [6]. In Ethiopia, scabies is also common especially during natural or manmade disasters such as flooding, drought, civil war and conflict, poor water supply and sanitation, and overcrowding living conditions [7, 8]. A study in Northern Ethiopia, Gondar town, among “Yekolo Temari” (Ethiopian orthodox church education attendants), revealed 22.5% scabies prevalence; however, another study conducted in southern Ethiopia revealed a prevalence of 5.5% among schoolchildren [9, 10].

The predominant symptom of scabies infestation is pruritus, which can be debilitating. Disruption of the skin’s protective barrier function promotes secondary bacterial infections, which can lead to additional, potentially life-threatening complications [11]. The main effect of scabies is debilitating itching, leading to scratching, which in turn is followed by the breakdown of the barrier function of the skin and complications due to bacterial infection, ranging from impetigo, abscesses, and cellulitis to more serious conditions such as septicemia and glomerulonephritis, leading to renal failure and rheumatic heart disease [12].

Transmission is influenced by social attitudes, migration, access to healthcare services, housing conditions, hygiene conditions, and crowding. Endemic scabies occurs with severe infestations, complications, and sequels, mainly in children [5]. It is reported that overcrowded living conditions, sleeping together, sharing of clothes, sharing of towels, poor hygiene practices, malnutrition, and travel to scabies outbreak areas are common risk factors for scabies [13]. The disease is transmitted through direct and prolonged contact with infested skin or rarely by using contaminated personal objects [14, 15]. It has a significant impact in terms of cost of treatment, absence at work or school, and psychological repercussions [16]. In schoolchildren, the infestation often spreads quite rapidly, owing to their close contact and overcrowding within the schools [6, 14, 15]. Additionally, this health threat has a negative impact on learning [17]. Sleep loss as a result of scabies-related itching is very common. Shame, restriction of leisure activities, and stigmatization are also encountered among scabies affected individuals [5].

Scabies has become a major public health problem, especially in low resource settings. There is a scarcity of evidence regarding scabies prevalence and associated risk factors among schoolchildren in our study area. Therefore, the aim of this study was to assess the prevalence of scabies and associated factors among students in primary schools in Dabat district, northwest Ethiopia. This study shed light on the burden of scabies and its prevention and control methods in Ethiopia.

## Methods

### Study design and population

**Fig. 1:**
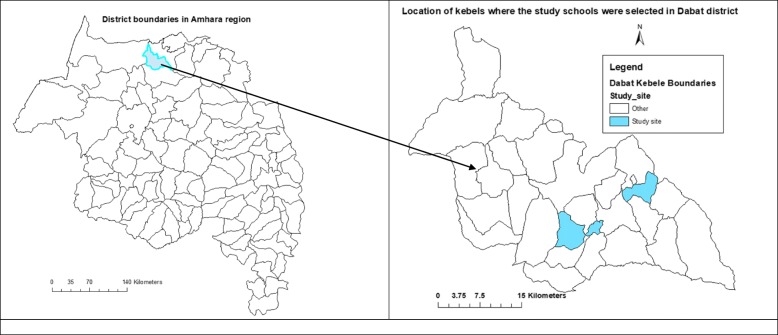
Map of the study area

The institutional-based cross-sectional study was conducted in Dabat district (Fig. 1) from May 1 to 15, 2018, to determine the prevalence of scabies and associated factors among schoolchildren. The study area is generally characterized by poor sanitation coverage, poor water supply and a high burden of parasitic infection [18].

### Sample size determination

The sample size was determined using a single population proportion formula with the following assumptions: margin of error 2.5%, proportion of scabies among schoolchildren 5.5% [8], 95% confidence interval, and design effect of 1.5 as in the absence of previous literature taking a design effect of 1.5 to 2.0 is suggested [19] and 10% of non-response rate to come up with a sample size of 527 respondents (schoolchildren). The actual calculated design effect taking the ratio of the standard error using a two-stage design to the standard error based on simple random sampling after the study completed was 1.42 which is closer to the design effect we used to calculate the sample size. Moreover, the rate of homogeneity calculated from the design effect and average number of individuals per cluster was 0.007 which shows that implementing the two stage sampling is best option.

### Sampling technique and procedure

A multistage random sampling technique was used to select study participants in participating public schools. We have used two stages to select the final study participants in this study. In the first stage, 8 primary schools were selected randomly from a total of 28 schools in Dabat district. Then, from each selected school, study subjects were selected proportionally to their size by simple random sampling technique.

### Diagnostic approach and operational definitions

#### Scabies

Scabies was diagnosed based on physical examination by experienced health officers. If lesions were observed during the physical examination on body surfaces such as interdigital spaces, hand, wrist, arm, elbow, axilla, leg, foot, abdomen, thorax, mamilla/perimammillar area, back, buttock, genital/inguinal area, and head (scalp/neck/face), they were reported as scabies [20]. Primary lesions were distinguished as macular, papules, crusted papules (if a tiny hemorrhage crusts), vesicles, and nodules [5, 20].

#### Source of water

Drinking water sources were categorized as “protected” if the community fetched water from protected springs, protected wells, or public taps and “unprotected” if the water source was river, unprotected springs, and unprotected wells. Drinking water supply service level was taken as “no access” and “basic access” if households had collected below 20 l per capita per day (l/c/d) and 20 l/c/d and above water, respectively.

#### Hand washing practice

Hand washing practice was taken as “good” if children washed their hands with soap before a meal, after defecation, after meals, before food preparation, after visiting the toilet, and after handling rubbish or animals.

#### Knowledge

Participants were asked to answer 14 knowledge-related questions about the nature, ways of transmission, prevention, and control measures of scabies. They were considered as having “good knowledge” if they had correctly answered the median and above the number of questions (9 to 14 out of 14 questions) and otherwise as having “poor knowledge.”

### Data collection tool and procedure

The data collection tool was prepared by reviewing different literature on similar studies [6, 8, 9, 17, 21]. The semi-structured, pre-tested Amharic version of the questionnaire was used to collect sociodemographic characteristics of students and their parents, knowledge of students about nature, prevention and control, and transmission methods of scabies, environmental and behavioral factors, school-based water, and sanitation system. The data was collected by eight BSc environmental health professionals. Two supervisors were also involved in monitoring data collection and checking the completeness of the questionnaires. Subsequently, a physical examination was independently undertaken by two experienced medical practitioners to diagnose scabies. Disagreements between the medical practitioners (health officers) were reconciled through discussion and consensus.

### Data processing and analysis

The data were entered using Epi-Info version 7 and analyzed using the SPSS statistical package for Windows, version 20.0. All assumptions for binary logistic regression were checked. To determine predictor variables for scabies, a binary logistic regression model was fitted and variables at a *p* value < 0.2 in the bivariable analysis were included in the multivariable analysis. Finally, variables found to be significant at a *p* value < 0.05 in the final model were declared as predictor variables. Crude odds ratios (COR) and adjusted odds ratios (AOR) with 95% confidence interval were reported in the result.

## Results

### Sociodemographic characteristics of students and their parents

A total of 494 study participants were included in this study with a response rate of 91.84%. The median (interquartile range (IQR)) age of the respondents was 12 years (10–14 years), and 46.7% (231/494) of them were aged from 11 to 15 years old. More than half 57.5% (284/494) of the respondents were male. Two hundred thirty-one (46.76%) of students’ father were illiterate by educational status. More than half (52.0%) of students had grade levels of 1 to 4. One hundred and four (21.1%) of students had one or more of their family infested by scabies (Table 1).

**Table 1:** Sociodemographic characteristics of students and their parents, Dabat district, Northwest Ethiopia, 2018 (*n* = 494)

Variables	Frequency	Percent
Sex		
Male	284	57.5
Female	210	42.5
Mother educational status		
Illiterate	267	54.05
Literate	227	45.95
Father educational status		
Illiterate	231	46.76
Literate	263	53.24
Location of school		
Rural	237	48.0
Urban	257	52.0
Children residence		
Rural	259	52.4
Urban	235	47.6
Age		
5–10	157	31.8
11–15	231	46.7
> 15	106	21.5
Family size		
1–5	272	55.1
> 5	222	44.9
Student grade level		
1–4	257	52.0
5–8	237	48.0
Living arrangement		
With both mother and father	378	76.5
Not with both mother and father	116	23.5
Family member with itching symptom		
Yes	104	21.1
No	390	78.9

### Environmental and behavioral factors

More than half (58.1%) of the parents of students got drinking water from protected sources. Nearly two thirds of students shared a blanket during bedtime with their family members, and a quarter of students (24.5%) had physical contact with scabies cases. Majority of students, 450 (91.1%) took a bath once per week, and 431 (87.25%) of students used water and soap/ash/endod (*Phytolacca dodecandra*) for handwashing (Table 2).

**Table 2:** Environmental and behavioral characteristics of students in Dabat district, northwest Ethiopia, 2018 (*n* = 494)

Variables	Frequency	Percent
Water source		
Protected	287	58.1
Unprotected	207	41.9
Accessibility of water source		
Accessible	378	76.5
Not accessible	116	23.5
Family member with itchy symptom		
Yes	104	21.1
No	390	78.9
Sleeping place		
On bed	479	97.0
On floor	15	3.0
Sharing blanket during bed time		
Yes	310	62.8
No	184	37.2
Ever had contact with scabies case		
Yes	121	24.5
No	373	75.5
Frequency of bath		
Once per week	450	91.1
Rarely	44	8.9
Frequently used hand washing material		
Water only	63	12.75
Water and soap/ash/endod	431	87.25
Knowledge of children		
Good	83	16.8
Poor	411	83.2

### Prevalence of scabies

The prevalence of scabies among schoolchildren was 9.3% (95% CI 5.66%, 12.94%). Among children with confirmed scabies cases, 65.22% had mild, 28.26% had moderate, and 6.52% had severe lesions. Eighteen (39.13%) of children with scabies encountered discrimination due to their status and 43.47% of children reported a sleeping problem due to frequent itching.

### Factors associated with scabies

Physical contact such as contact with scabies case, presence of family member with itchy signs, and the use of only water for hand washing were among the factors contributing for scabies infestation transmission.

Students who ever had contact with a person having itchy symptom 2 weeks prior to the study were at a higher chance of being infested by scabies (AOR = 2.66, 95% CI 1.21, 5.83). The likelihood of being infested by scabies was 4.76 times (AOR = 4.67, 95% CI 2.20, 10.28) more likely among students with at least one family member having itching lesion than students who had no family member with itching lesion.

Students who used only water for handwashing were 4.38 times more likely to be infested by scabies than students who washed their hands with water and soap/ash/endod (AOR = 4.38, 95% CI 1.78, 10.76).

Other factors that are associated with scabies prevalence include knowledge and behavior of children and environmental factors. The study also showed that students with grade levels of 1 to 4 were 3.91 times more likely to develop scabies (AOR = 3.91, 95% CI 1.69, 9.05). The odds of being infested by scabies among students who had bath rarely was 3.54 times (AOR = 3.54, 95% CI 1.36, 9. 25) more than students who had a bath at least once a week.

Lastly, the sociodemographic characteristics of both the children and their parents, such as location of the school, father’s educational status, and students’ grade level, play pivotal role in the transmission of scabies.

The odds of being infested by scabies among study participants who attended a school located in a rural area was 2.99 times (AOR = 2.99, 95% CI 1.33, 6.71) more than their counterparts who attended in schools located in an urban area. Students whose father were illiterate were 5.11 times more likely to develop scabies than students who had a literate father (AOR = 5.11, 95% CI 2.25, 11.58). Schoolchildren who do not live with both of their parents were 2.49 times at higher risk of infestation by scabies than those who live with both of their parents (AOR = 2.49, 95% CI 1.02, 6.06) (Table 3).

**Table 3:** Multivariable analysis of predictors for scabies among students in primary school in Dabat district, northwest Ethiopia, 2018 (*n* = 494)

Variables	Scabies		COR with 95% CI	AOR with 95% CI
	Yes	No		
Location of school				
Urban	31	226	1	
Rural	15	222	2.03 (1.07, 3.86)	2.99 (1.33, 6.71)**
Mother’s education				
Illiterate	31	236	1.86 (0.98, 3.53)	0.88 (0.39, 1.98)
Literate	15	212	1	
Father’s education				
Illiterate	30	201	2.30 (1.22, 4.35)	5.11 (2.25, 11.58)***
Literate	16	247	1	
Students’ grade level				
1–4	34	223	2.86 (1.44, 5.66)	3.91 (1.69, 9.05)***
5–8	12	225	1	
Students’ living arrangement				
With both parents	28	350	1	
Not with both parents	18	98	2.30 (1.22, 4.32)	2.49 (1.02, 6.06)*
Frequency of bath				
At least once per week	35	415	1	
Rarely	11	33	3.95 (1.84, 8.49)	3.54 (1.36, 9.25)*
Sleeping place				
On bed	42	437	1	
On floor	4	11	3.78 (1.15, 12.40)	2.12 (0.52, 8.65)
Ever had contact with a person with itching symptom				
Yes	25	96	4.36 (2.34, 8.14)	2.66 (1.21, 5.83)*
No	21	373	1	
Family member with itchy signs				
Yes	25	29	5.56 (2.96, 10.43)	4.76 (2.20, 10.28)***
No	21	369	1	
Frequently used hand washing material				
Water only	15	48	4.03 (2.03, 8.00)	4.38(1.78, 10.76)*
Water and soap/ash/endod	31	400	1	

**** significant at *P*-value < 0.001 ** significant at *P*-value < 0.01, * Significant at *P*-value < 0.05, *COR* Crude Odds Ratio, *AOR* Adjusted Odds ratio, *CI* Confidence interval

## Discussion

Data on prevalence and associated risk factors about scabies infestation in schoolchildren provide valuable information to serve as a basis for methods of prevention and control and therapeutic services. In the current study, the overall prevalence of scabies among schoolchildren in Dabat district was 9.3%. This is higher than the prevalence of scabies reported among schoolchildren in Ibadan, Nigeria, with a prevalence of 4.8% [22], in a study conducted on pediatric dermatology clinic patients in Kuwait 3% [23], in a study conducted among primary schoolchildren in Egypt with a prevalence of 4.4% [6], and among schoolchildren in Nigeria Katanga with a prevalence of 2.9% [24], and it is similar with previous study done among schoolchildren in southern Ethiopia with a 5.5% prevalence [10]. On the other hand, higher prevalence rates of scabies were reported among Indian primary schoolchildren living in a slum of Kolkata with a 39.42% prevalence [25], among children in four boarding schools in Cameroon with a 17.8% prevalence of scabies [26] and among “Yekolo Temaris” in Gondar, northwest Ethiopia, with a prevalence of 22.5% [9]. The difference in prevalence rates could be attributed to the density of population, overcrowding, poverty, level of health services, variation in sociodemographic characteristics of the study population, and climatic variability. High rates of scabies are usually found in communities and settings where overcrowding and poverty are highly prevalent [14, 15]. The difference in the prevalence rates might also be attributed to the time and season of the study and weather and environmental condition of the study area [27].

Several modifiable and non-modifiable physical contact-related, knowledge, environmental, and behavior as well as sociodemographic risk factors were identified in this study. The presences of having a family member with a history of itchy skin lesions and contact with a person with a history of itchy skin lesion were significantly associated with scabies infestation. This finding is aligned with the results of many previous studies [6, 28, 29]. This might be due to the fact that family members spend sufficient time together for the scabies mite to be transferred to the healthy member of the family and the most efficient mechanism of scabies transmission is through direct skin contact reflecting the fundamental role of physical contact in person-to-person transmission. The history of itching in several family members over the same period is almost pathognomonic [30]. However, the lack of history of itching in family members does not preclude scabies.

The frequently used hand washing material was a significant predictor of scabies infestation among schoolchildren in this study. Those students who have used hand based sanitizers like soap, ash, etc., had a better chance of preventing themselves from scabies infestation. This might be due to the fact that the sanitizers will get rid of the scabies mites easily. The susceptibility of the mites to different hand washing sanitizers requires further study. The children’s behavior and environmental factors also contribute to the scabies transmission. Lower-grade primary schoolchildren were at higher risk of scabies infestation as compared with higher grade students. Frequency of bath was also associated with a risk of scabies. Those study participants who take a body shower at least once per week had a higher chance of preventing scabies as compared to their counterparts who do not take bath frequently. This was in line with a study conducted by Emmanuel et al. among children and adolescents living and studying in Cameroonian boarding schools [26]. This might be because students at first cycle primary schools have lesser control over their personal hygiene and environmental sanitation. They also have a higher chance of contact as they spend much of their time playing with their friends. The spreading of human scabies is linked with poor personal hygiene [14, 15]. Therefore, it is possible that the younger students, who are of lower educational level than their counterparts, are less aware of personal hygiene rules to adopt especially when living with others, being, therefore, more prone to be infested.

There was no statistically significant difference in the prevalence of scabies among male and female students in the current study. This result was in agreement with the results of other studies [6, 24, 31]. Being male was observed to be a risk factor in other studies [17, 26, 32]. These differences might be attributed to other social determinants of health in each society or country.

The current study revealed that the prevalence of scabies was higher among children from rural schools as compared with those from urban schools. This finding is supported by many previous studies from different countries [6, 21, 32]. This might be due to the fact that rural areas are predominantly characterized by overcrowding, the lesser tradition of personal hygiene such as taking a bath beside the decreased level of health education, poverty, bad behavioral habits such as sharing clothes and bed linen with others, and dealing with animals.

The prevalence of scabies in the present study was higher among children whose fathers were illiterate as compared with among those whose fathers were literate. These results are in line with several previous studies [6, 33–35]. This might be because illiteracy of household head was a superb predictor of the presence of scabies in developing countries. The educational statuses of the fathers are determinants of social class, and as the paternal level of education improves, the family income and hence health consultation-seeking behavior also improves reducing frequent scabies infestation. Schoolchildren who do not live with both of their parents were 2.49 times at higher risk of infestation by scabies than those who live with both of their parents. This might be due to the fact the children who lives with both of their parents will get necessary level of support easily than those who live with single parents.

Students who were infested by scabies reported several problems including being socially excluded, insulted, the difficulty of writing and attending classes for the sleeping problem at nights because of intense itching, and lack of sufficient time for sleeping.

Some limitations of our study include the absence of dermoscopy and/or skin scrapings/microscopy, although they are operator-dependent and have relatively low sensitivities. Our diagnosis was entirely based on clinical assessment independently conducted by two experienced health officers. Second, the inherent nature of cross-sectional design does not establish a cause-effect relationship. Third, our findings might not be generalized to the entire population of Ethiopian primary school students given that we worked only in eight schools at one season. Because we have done the study at an institutional level, some housing-related factors such as wealth index were not covered in the current study.

## Conclusion

The prevalence of human scabies among schoolchildren in Dabat district northwest Ethiopia was high. The school localization, first cycle level of education, paternal educational status, frequency of taking a bath, and contact with a person having itchy lesions, presence of a family member with itchy lesion, and type of frequently used hand washing material were the independent factors significantly impacting the occurrence of scabies. Special attention should be given to students at first cycle education as they are at the highest risk of infestation. Improving the hygienic behavior of students and reducing contact with scabies and treatment of the entire family in case of the infestation should be encouraged. Personal hygiene rules should be taught to students, with special emphasis among first cycle students. More studies at the community level to better understand additional factors driving the infestation by scabies and its impact on the quality of life of the children affected should be undertaken.

## Data Availability

Data will be made available upon requesting the primary author.

## Abbreviations

AOR: Adjusted odds ratio
CI: Confidence interval
COR: Crude odds ratio
IQR: Interquartile range
SPSS: Statistical Package for Social Sciences
